# Offsetting anthropogenic carbon emissions from biomass waste and mineralised carbon dioxide

**DOI:** 10.1038/s41598-020-57801-5

**Published:** 2020-01-22

**Authors:** Nimisha Tripathi, Colin D. Hills, Raj S. Singh, Jamuna S. Singh

**Affiliations:** 1 Indo-UK Centre for Environment Research and Innovation, University of Greenwich, ME4 4TB London, UK; 2 Indo-UK Centre for Environment Research and Innovation, CSIR-Central Institute of Mining and Fuel Research, Dhanbad, India; 3 Indo-UK Centre for Environment Research and Innovation, Banaras Hindu University, Varanasi, India

**Keywords:** Environmental impact, Composites

## Abstract

The present work investigates biomass wastes and their ashes for re-use in combination with mineralised CO_2_ in cement-bound construction products. A range of biomass residues (e.g., wood-derived, nut shells, fibres, and fruit peels) sourced in India, Africa and the UK were ashed and exposed to CO_2_ gas. These CO_2_-reactive ashes could mineralise CO_2_ gas and be used to cement ‘raw’ biomass in solid carbonated monolithic composites. The CO_2_ sequestered in ashes (125–414 g CO_2_/kg) and that emitted after incineration (400–500 g CO_2_/kg) was within the same range (w/w). The CO_2_-reactive ashes embodied significant amounts of CO_2_ (147–424 g equivalent CO_2_/kg ash). Selected ashes were combined with raw biomass and Portland Cement, CEM 1 and exposed to CO_2_. The use of CEM 1 in the carbonated products was offset by the CO_2_ mineralised (i.e. samples were ‘carbon negative’, even when 10% w/w CEM 1 was used); furthermore, biomass ashes were a suitable substitute for CEM 1 up to 50% w/w. The approach is conceptually simple, scalable, and can be applicable to a wide range of biomass ashes in a closed ‘emission-capture’ process ‘loop’. An extrapolation of potential for CO_2_ offset in Europe provides an estimate of CO_2_ sequestration potential to 2030.

## Introduction

As the world’s population increases to 11.2 billion by the year 2100^1^, the available resources to meet desired living standards must increase accordingly. By way of example, the supply of energy is projected to increase at an annual rate of 1.6%/yr, to 2030^2^. Due to the increasing demand for energy, the Organisation for Economic Co-operation and Development (OECD) expect greenhouse gases (GHGs) to increase by 50% by 2050, and possibly to 750ppm by 2100, if no adequate management options are sought^3^.

The growing world population will drive the intensification of agricultural industrial activities, and as a consequence, larger quantities of waste/residues from both harvestable yield and non-harvestable biomass can be expected to be produced. The current global annual generation of all biomass waste including animal waste, is in the order of 140 Gt^2,4^, and when their disposal, utilisation and management are inappropriate, adverse environmental impacts arise.

In developing countries, most biomass residues are left in the field to decompose naturally or are burned in the open; impacting surface water and the atmosphere. By way of example, 1 tonne of landfilled dry, ash-free wood produces 0.73 ton CO_2_^5^, whereas 1 tonne of fuel wood produces approximately 1.4 t or CO_2_^6^.

Biomass burning contributes about 18% of total global emissions^7,8^, with 70% of this arising from use as domestic fuel, primarily by poor and agrarian communities^9^. The IEA projects that forestry and agriculture wastes will continue to increase^10^, with Asia and North America accounting for two-thirds of biomass wastes arising from crop production^11^.

If biomass residues have potential for other uses, their displacement should follow *the* “waste management hierarchy”, namely: prevention, re-use, recycling (including composting), energy recovery, and (only when no other options are available) disposal^12^. Low energy, low carbon management solutions that valorise waste are, therefore, a preferred option.

The valorised products from biomass ashes have potential to be significantly carbon negative in a ‘closed loop’ manufacturing process^13^. Biomass waste to energy plants emit 47 Mt CO_2_ each year^14^, and the ashes generated tend to be reactive to CO_2_ to a lesser or greater degree. By using ‘point source’ CO_2_ or flue gas capture in manufactured products made from ashes, this circular economic activity will reduce solid and gaseous emissions, landfilling and the extraction of virgin stone.

Concrete is the second most consumed material on Earth after water, with approximately 3.27 Gt currently produced, rising to 4.83 Gt by 2030^15^. Andrews^16^ estimates that 39.3 ± 2.4 Gt CO_2_ were emitted by the cement industry during the period 1928–2016, with 90% being generated since 1990. As clinker production is currently growing at about 2.5% pa, generating 5–7% of total global CO_2_ emissions, technologies that reduce our reliance on cement or materials that can be used as a substitute are timely. Current estimates of the amount of CO_2_ emitted by cement production vary between 500–900 kg CO_2_/t cement produced, depending on the source of fuel and analysis methodology^17,18^.

As stated, a balance between positive and negative emissions is required in quest of meeting the commitments of 195 nations to hold the increase in the global average temperature to below 2 °C above pre-industrial levels under the Paris Agreement^19,20^. Technologies that transform waste and CO_2_ into products (e.g., construction materials, plastics and fuels) are now being developed and commercialised. The capture of CO_2_ from flue gas or the atmosphere as feedstock in value-added products is referred to as carbon capture and utilisation (CCU), including the mineralisation of CO_2_ in combination with solid waste^21,22^.

To date, the valorisation of biomass residues and their ashes has been largely overlooked, despite being ubiquitous, plentiful and rich in carbon. As developing countries, such as India, seek alternative resources to meet their infrastructure growth and emissions reduction needs^23^, valorised, sustainable biomass waste-based products may have a part to play.

World demand for construction aggregates will rise 5.2 percent annually to 51.7 Gt in 2019^24^. In the UK for example, the construction industry accounts for 60% of all raw materials consumed^25^ and thus, low carbon waste-based construction materials are already available in the market.

The diversion of biomass wastes into construction products is being investigated in Europe^26^, as virgin materials resources are under increased pressure. However, the use of biomass ash with mineralised CO_2_ has not been explored for the manufacture of construction materials. The opportunity to combine biomass (waste) ash from energy plants with waste CO_2_ gas in an innovative CCU-based treatment step is another approach in the drive towards a sustainable materials supply chain for the construction industry.

In the present work, which is part of a wider study, we investigate the re-use potential of biomass waste for use in cement-bound construction products. Herein, we exploited the self-cementing property of biomass ashes when exposed to waste gaseous CO_2_. We applied a CO_2_ mineralization method to develop biomass waste (and their ashes) - based construction materials. As biomass ashes can self-harden as CO_2_ is mineralised, they could be used to replace cement in carbonated biomass-based construction materials; and potentially offset ‘carbon’. The utilisation of CO_2_ in waste-derived valorised products could help maintain a balance between point source emissions to the atmosphere and CO_2_ sequestrated in mineralised, valorised products.

## Methods

### Characterisation of biomass wastes

Biomass waste originating from agricultural and forestry activities, including wood, fruit peel, nut shell and fibre were sourced in India, Africa and the UK. These wastes were combusted into muffle furnace at 900 ± 25 °C. The temperature was raised gradually from 200 to 500 to 900 °C. Our primary aim was to produce a ‘mineral’ concentrate by fully combusting the biomass residues examined, and we used an uncontrolled heating rate to achieve this. The furnace specification stated that the tolerance was ±25 °C. The temperate was held at 900 °C for 4 h to ensure the full combustion of the biomass.

The resulting ashes were examined for their physical (e.g. particle size, bulk density, and ash content) and chemical (total carbon, elemental and phase) composition. The particle size distribution of ashes was measured by laser diffraction analysis (Malvern Mastersizer MS2000) and bulk density by loose compaction in cylindrical holders (expressed as kg/m^3^). Total carbon was analysed by the CHN analyser (FLASH EA 1112 Series), and the elemental composition was determined by X-ray fluorescence spectrometry (Philips LW1400 and XRFWIN software).

The BET surface area of biomass ashes was analysed with a surface area analyser (Micromeritics Gemini V2.00) using nitrogen adsorption measured as a function of relative pressure.

The biomass ashes were tested for their reactivity with CO_2_ under controlled moisture (20%) and pressure (~2 bars). The ashes were exposed to pure CO_2_ over four different cycles in a closed pressurised carbonation chamber. The first three cycles extended to one hour each, whereas the fourth cycle extended to 24 hours. The uptake of CO_2_ in ashes was determined by weight gain (% w/w).

### Preparation and characterisation of products made from biomass wastes

(i) Based on the mineralogy and CO_2_-uptake of biomass waste ashes, small cylindrical ash-only monolithic samples (7 mm × 7 mm) with 10–20% moisture were cast.
(ii) These monoliths were exposed to pure CO_2_ for 24 hrs and evaluated for embodied carbon.
(iii) CO_2_-reactive biomass ash was mixed with raw biomass and cast in larger (3.4 cm × 3.4 cm) cylinders. These cylinders also included biomass waste in combination, with Portland cement, CEM 1 (plus fine sand as a mineral filler) at 10–20% moisture to assess biomass ash as a substitute for CEM 1. Cylinders were exposed to pure CO_2_ for one week.

The CO_2_ uptake by both types of cylinders was calculated as CO_2_ equivalent from the total carbon analysed by CHN analysis. The strength of these monolithic products was evaluated by applying a force until the cylinders failed. The strength was calculated by using the Eq. 1:

1 $${\sigma }_{c}=\frac{2.8\,{F}_{c}}{{{\rm{\pi }}\text{dm}}^{2}}$$

where ${\sigma }_{c}$ is the compressive strength in megapascals, F_*c*_ is the fracture load in kilonewtons, A*m* is the mean area of the cylinder, and d*m* is the mean diameter of the cylinder.

For each sample, 5 cylinders were examined, and the average strength was calculated. The three axes of each cylinder were measured using digital callipers, and the load at failure determined (Mecmesin MFG250).

### Assessment of CO_2_ uptake in valorised biomass products

The biomass ashes and resultant carbonate-cemented products were investigated by X-Ray diffractometry and back-scattered electron microscopy.

(i) X-Ray Diffractometry: The biomass ashes without and with CO_2_ exposure were analysed with a Siemens D500 diffractometer, fitted with a Siemens K710 generator using 40 kV voltage and 40 mA current, between 5–65° 2θ. The interpretation of diffractograms utilised DIFFRAC^*plus*^ EVA software (Bruker AXS).
(ii) SEM/EDAX analysis (JEOL JSM-5310LV, Oxford Instruments Energy Dispersive Spectrometer- EDAX) was performed upon polished resin blocks containing carbonated and un-carbonated biomass products.

## Results

### Reactivity of biomass ash with CO_2_

A range of biomass residues (including wood chips and shavings, nut shells, fibre, and fruit peel) obtained from Asia, Europe and Africa, were collected and characterised (Flash EA 1112 Series, CHN analyser). Following, they were thermally degraded, and the ‘mineral’ rich ashes were exposed to CO_2_ gas under controlled, static conditions. Four cycles of CO_2_ exposure were investigated (three 1-hour cycles, followed by a 24 hr fourth cycle of treatment) (see Table S2 in Supplementary Materials). The CO_2_ uptake, %w/w, was determined as a CO_2_ equivalent, derived from the total carbon observed in the ashes after each treatment step. A gradual increase in CO_2_ uptake was observed from the first to last CO_2_-exposure cycles, demonstrating that biomass ashes can be used to mineralise CO_2_ gas (via the formation of calcium carbonate cement) and thereby encapsulate the ‘raw’ biomass in solid carbonate-cemented monolithic composites.

An examination of ash mineralogy by X-ray diffraction (Siemens D500 diffractometer and DIFFRAC^*plus*^ EVA software- Bruker AXS) was used to examine the mineral phases present in the biomass ash and its carbonated counterpart. The results showed that the calcium oxide (CaO) in biomass ash reacted with the CO_2_ to produce calcium carbonate. This mineral induced strength development through carbonate-cementation.

**Figure 1:**
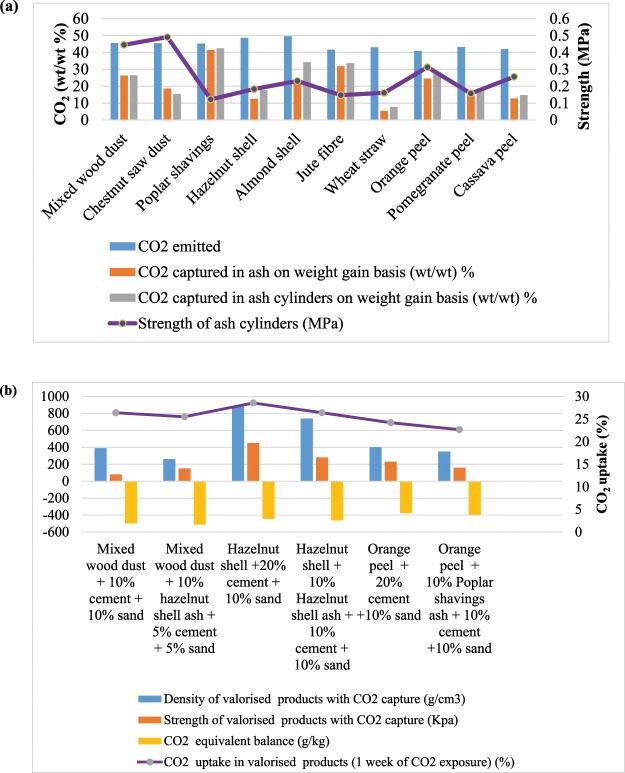
(**a**) CO_2_ (equivalent) emission and capture potential (w/w%) of biomass residue ashes and ash cylinders, and their compressive strength in MPa (secondary vertical axis). **(b)** CO_2_ balance (g/kg), density (g/cm^3^), strength (KPa) and CO_2_ uptake (secondary vertical axis) (%) of valorised products from raw biomass with CO_2_-reactive ashes.

Incinerated biomass emitted 400–500 g CO_2_/kg, but when the ashes were carbonated, they sequestered –49-414 g of CO_2_/kg after 24 hrs of exposure to this gas (Fig. 1a; Table S3 in Supplementary Material). On a weight for weight basis, the CO_2_ ‘emissions’ and ‘uptakes’ were within the same range, however, the mass lost during thermal decomposition is far greater than that gained during carbonation of the ash.

Some woody, fibre and nutshell-ash residues were observed to combine with significant amounts of CO_2_. Small cylindrical ‘ash-only’ monolithic samples (7 mm × 7 mm) were cast and exposed to CO_2_ for 24 hrs. The amount of CO_2_ up-taken varied between 147–424 g equivalent CO_2_/kg ash upon carbonation (Fig. 1a). These results show that all the biomass ashes investigated were CO_2_ reactive and produced mineral carbonates, which have potential to act as a carbonate-able binder, or as a partial substitute for hydraulic cements in certain applications involving Portland cement, CEM 1.

Following these findings, biomass ash was mixed with raw wood chips, nut shells or fruit peel and larger (3.4 cm × 3.4 cm) cylinders were cast and exposed to CO_2_. Strength was developed through carbonate cementation as previously observed. In addition, biomass ashes were investigated in combination with CEM 1 and fine sand as a mineral filler (Fig. 1b). Here, ash was used as a partial substitute for the cement during the production of the monolithic composites.

### Impact of particle size and surface area

The biomass ashes had higher BET surface area than their raw counterparts (Table 1). The mineralisation of CO_2_ in biomass ashes is promoted by small particles as they have a higher surface area for reactions to proceed^27,28^. The addition of wood, nutshell and fruit peel-derived ashes to raw biomass enabled carbonation-induced cementation to produce a hardened composite.

**Table 1:** Particle size and BET surface area of raw biomass and their ash.

Biomass waste	Biomass ash particle size (mm)	Surface area (m^2^/g)	
		Raw biomass (m^2^/g)	Biomass ash (m^2^/g)
Mixed wood chip	0.26	1.94	6.23
Poplar bark shavings	0.16	1.66	6.72
Chestnut sawdust	0.24	0.92	4.87
Hazel nut shell	0.18	0.9261	2.61
Almond shell	0.24	0.36	1.43
Jute fibre	0.45	1.42	2.71
Straw (wheat)	0.11	1.35	2.62
Cassava	0.37	0.67	1.08
Pomegranate	0.15	0.28	1.93
Orange	0.48	0.93	1.24

Possan *et al*.^29^ reported that CO_2_ taken/used by mixed wood/coal ashes is largely regulated by surface area. However, particle size is not always the limiting factor for CO_2_ uptake. The findings of Nam *et al*.^30^ with municipal solid waste ash, showed the amount of CO_2_ sequestered increased as particle size decreased. This may well be valid for ashes with a similar chemistry, but where the amount of calcia varies in a feedstock, as seen in this work, particle size/surface area may be a secondary consideration.

The use of biomass residues in novel applications is of interest. Plant biomass, including dry miscanthus has been used for heavy metal removal from waste water^31^, but as a management strategy, large quantities of materials need to be utilised, such as in construction applications. The combination of biomass waste with cementitious binders to produce building materials has seen limited practice around the world^32,33^. Plant fibres derived from flax, hemp and straw are used to improve the mechanical properties of cement-based composites, but their durability performance is questionable. This results partly from the humid high pH environment (within the cemented product), as fibrous materials degrade via lime crystallisation and dissolution of cellulose and hemicellulose, and certain lignins^34–37^. Thus, the processing of biomass waste with mineralised CO_2_ as calcium carbonate may be expected to produce cemented product with a more favourable pH environment of <10^38^, thereby enhancing the durability performance of these fibrous residues.

The combined use of CEM 1 and ash at the different proportions up to 50% substitution was investigated. A comparison of the carbon dioxide ‘footprint’ of products enabled the embodied carbon in the ash-bound composites to be evaluated as a potential off-set to the use of CEM 1 (which is high in embodied carbon at ca. 670 kg eqCO_2_/t^39^).

The calculation of embodied CO_2_ involved all the materials employed, and the carbon dioxide gas uptaken showed the monoliths to be carbon negative, even when 10% w/w CEM 1 was used (Fig. 1b). Furthermore, the strength developed by the use of ash and a carbonation step were comparable to those of low density hydraulically-bound products used in construction applications^40^.

The major and minor elements including Ca, Fe, K, Mg, P, Na and Si in biomass were identified by X-ray fluorescence spectroscopy (XRF) (Philips LW 1400, with WIN Software) (see XRF results in Supplementary Material Table S2a–c). Selected key elements in biomass ashes are generally found in decreasing order of abundance as Ca > K > Si > Mg > Al (see Vassilev *et al*., for more information)^41^. The authors also reported a negative relationship between calcium and ash content in individual species of wood. We also observed a similar trend in wood (unpublished study of authors) and agricultural biomass. However, some of the soft peel residues had a high calcium and high ash content, which can be ascribed to their complex heterogeneous nature. For example, citrus fruit peel including orange has a high calcium content, and this seems to be a general finding with citrus fruit^42^.

The carbonation of biomass ashes was confirmed by X-Ray diffraction by the presence of calcite (Table 2; Fig. S1a–c in Supplementary Material). However, in some samples the presence of Ca(OH)_2_ and CaO indicated that complete carbonation was not always achieved.

**Table 2:** Key phases in uncarbonated and carbonated biomass ashes of three types.

Phases	Mixed wood dust		Nut shell		Wood shavings	
	Uncarbonated	Carbonated	Uncarbonated	Carbonated	Uncarbonated	Carbonated
Portlandite	√	√		√		
Periclase		√	√	√		
Calcite		√		√		√
Monohydrocarbonate		√		√		
Calcium oxide			√	√		
Calcium hydroxide					√	√

**Figure 2:**
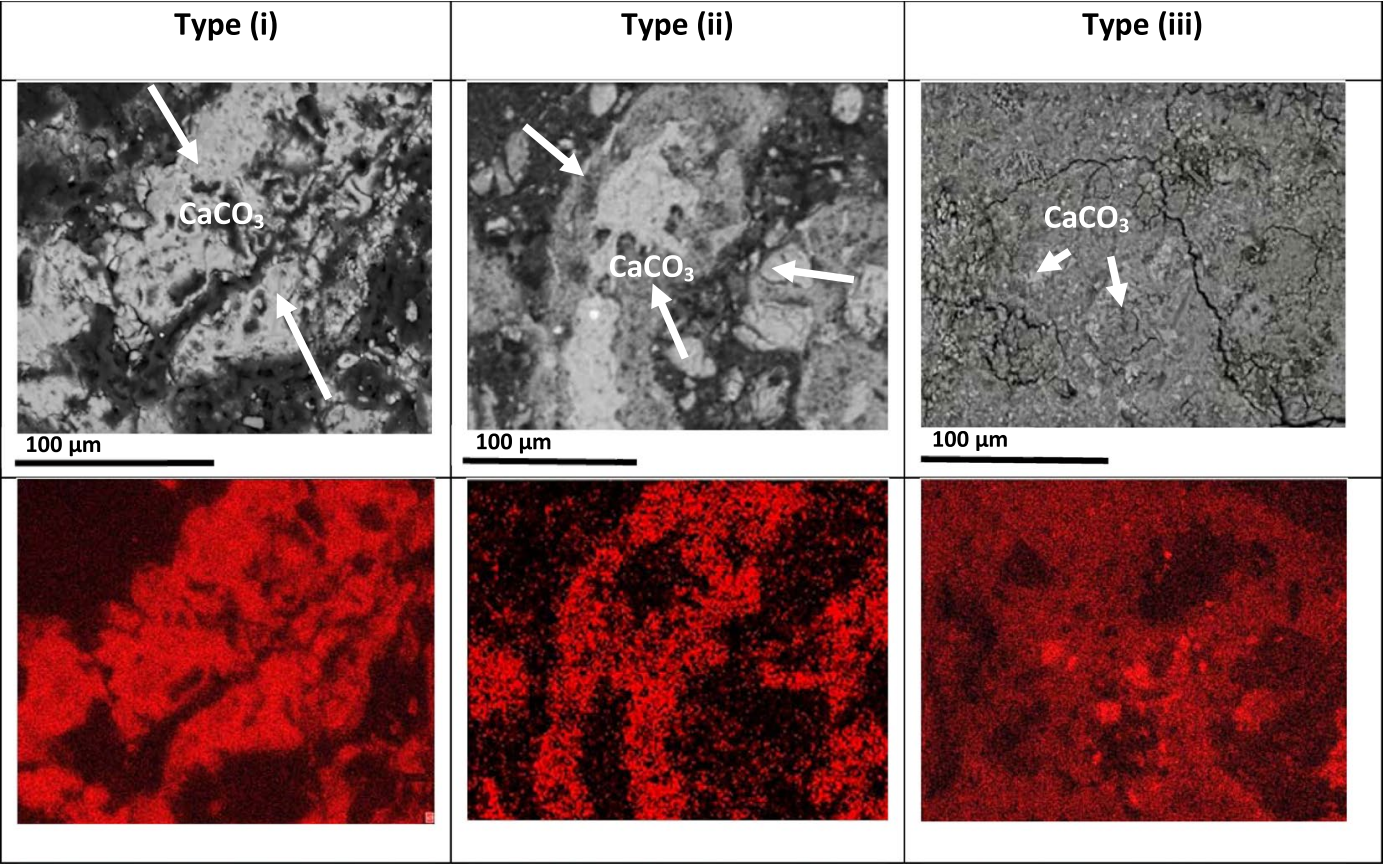
Back Scattered Scanning Electron Micrographs of CCU-treated ash cylinders: (Type i) - Mixed wood ash, showing relict-planty structures enveloped by massive carbonated precipitates; (Type ii)- Nut shell-derived ash cemented by interstitial carbonate; (Type iii)- Wood ash, with dispersed, discrete precipitates of carbonate. This ash was hygroscopic, displayed mircocracking and low strength (Note: Image 1 is taken at a slightly higher magnification for clarity).

Back scattered electron microscopy (Jeol JSM-5310 LV; Oxford Instruments EDS) was used to examine the microstructure of carbonated ashes and the distribution of key elements. The element maps obtained for Ca confirmed the presence of calcium carbonate, which gave rise to 3 distinct microstructures (see Fig. 2). Figure S2 in Supplementary Material gives the EDS spectra taken from the three carbonated samples. The 3 distinct microstructures observed comprised:

(i) Amorphous precipitates of carbonate often associated with relict (biomass) structures; forming a continuous cementing phase. Some of the relict structures were completely filled with carbonate. This microstructure was associated with well-cemented/hardened monolithic samples.
(ii) A more discrete distribution of carbonate precipitates occurring mainly on the surface of relict structures, especially at the point of contact between individual ash particles. These observations were also associated with well-cemented monolithic specimens.
(iii) Isolated, finely divided carbonate precipitates (typically ≤1 µm), distributed throughout the ash, with occasional agglomerations of approximately 5 µm in size. This microstructure was associated with lower-strength monolithic specimens.

Figure S3(a–c) in Supplementary Material gives further examples of the microstructure of carbonated ash monoliths/cylinders observed together with their element maps, showing the spatial distribution of specific elements.

**Figure 3:**
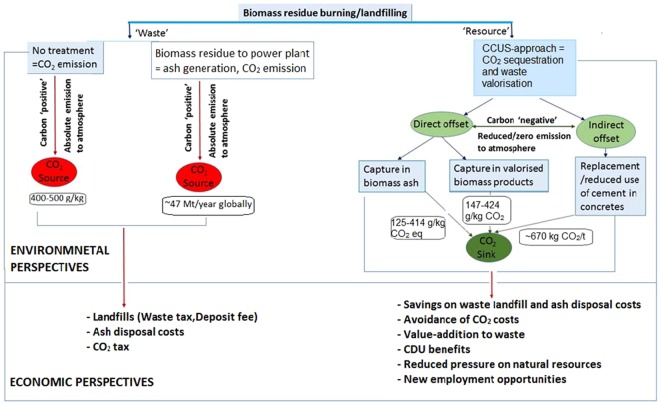
CO_2_ emission from burning biomass residues and offset pathways through carbon capture utilisation and storage (CCUS)-treatment.

Our study has clearly demonstrated that the mineralisation of CO_2_ gas in biomass-based monolithic composites is possible, and that even when used as a 50% substitute for CEM 1, meaningful quantities of carbon can be mineralised. The indicative carbon savings that can be realised on a broader scale are illustrated in Fig. 3. It should be noted, however, that composite materials using selected biomass ash as a substitute for hydraulic cement will require rigorous selection and independent testing to ensure compliance with international materials-related standards before the approach outlined could be adopted commercially.

Our findings also suggest there may be further added value to be gained as it is possible to extract, for example, proteins and fibres from biomass, before these residues are ashed and used as a carbonate-able binder. This particular work, which is in progress, will be reported separately.

## Discussion: A way forward

The utilisation of CO_2_ transformation technologies ideally involves stripping CO_2_ from a point source, or from the atmosphere before ‘storing’ it in a mineralised product. Our work shows this could be achieved by combining ‘raw’ biomass with *their* CO_2_-reactive ashes in a substitute for a hydraulic cement. The use of biomass ash in this way could help developing countries manage waste more effectively and reduce environmental impacts, whilst also providing for additional resources of building materials.

As an illustration of this potential, e.g., India is an emerging agro-based country with 159 M ha of arable land supporting ca. 800 Mt of agricultural/horticultural production, with 500–550 Mt/yr of waste arising^7,34^. There are significant surplus agricultural residues (raw biomass), estimated between 84–141 Mt per annum, with a further 90–140 Mt of ash, generated by burning on farm^7,43^.

As the Indian construction industry accounts for nearly 65% of total infrastructure investment, generating 11% of GDP (10640 billion INR, in 2016–17)^44,45^, the demand for construction materials in India is considerable. The use of aggregates alone is anticipated to reach 5 Gt by 2020^46^.

Similarly, significant agricultural residues are generated in Europe. Approximately, 276 Mt/p.a. of residues are generated from major cereal and oil-based crops^47^. From our studies, we can assume that 70% of these residues produce CO_2_-reactive ashes. Thus, if the average ash content is 5% (w/w dry weight) of that burned, and the CO_2_ reactivity is of the order of 10% (w/w dry weight) there is potential to mineralise about 1.0 Mt of CO_2_ in 10 Mt of ash in Europe. Furthermore, these reactive ashes can be used to carbonate-cement the remaining 30% non-reactive biomass residues (utilising 83 Mt of raw residues from cereal and oil crops) into useful monolithic products. The available residue from these cereal and oil crops in Europe is projected to be 340 Mt by 2030, which will further mineralise 1.2 Mt CO_2_ directly in ash and also indirectly in raw residues replacing cement. On a global scale, the projections for 2050 indicate an increase in demand for all biomass, with a larger proportion from agricultural residues being used to produce energy^48^.

A successful CCU-based treatment of biomass wastes, therefore, has potential to contribute to meeting this demand, especially if the residues can be used to replace carbon intensive materials, such as hydraulic cements (which may contribute 5% of the annual man-made carbon emissions worldwide^40,49^.

Our study indicates that a significant carbon positive ‘source’ of waste can be converted into a carbon negative ‘sink’, whilst producing products with potential value. The utilisation of biomass waste and their ashes combined with mineralised CO_2_ could help close the ‘emission-capture’ process ‘loop’, whilst also providing a sustainable management route for residues that have adverse environmental impacts. This method is conceptually simple, scalable, and potentially applicable to a wide range of biomass ashes produced in energy plants.

A diverse range of high-volume biomass residues appear to be suitable for processing with CO_2_ gas that would otherwise be emitted to atmosphere. The possibility of burning biomass for energy and then trapping the CO_2_ generated in a mineralisation step appears feasible. Thus, the processing of biomass waste via thermal destruction coupled with CO_2_ capture and a carbonation step, will reduce the environmental impacts and pestilence associated with these wastes, and help preserve virgin resources.

## Supplementary information

Supplementary material.

## Data Availability

Data is available as specified in the cited references. Data on research findings from this work is available in tables and figures.
