# Supplementary material for: Offsetting anthropogenic carbon emissions from biomass waste and mineralised carbon dioxide
Source: Sci Rep. 2020 Jan 22;10:958. doi: 10.1038/s41598-020-57801-5 (PMC6976572; doi:10.1038/s41598-020-57801-5)
Supplement: Supplementary file 1 — Supplementary material. [file 41598_2020_57801_MOESM1_ESM.pdf]

Offsetting anthropogenic carbon emissions from biomass waste and mineralised carbon dioxide

Nimisha Tripathi[1*], Colin D Hills[1*], Raj S Singh[2] and Jamuna S Singh[3]

Indo-UK Centre for Environment Research and Innovation

[1]University of Greenwich, ME4 4TB. UK

[2]CSIR-Central Institute of Mining and Fuel Research, Dhanbad. India

[3] Banaras Hindu University, Varanasi.India

*n.tripathi@greenwich.ac.uk; c.d.hills@gre.ac.uk

Table S1: Equivalent oxide composition of raw biomass (%w/w)

Biomass	Na_2O	MgO	Al_2O_3	SiO_2	P_2O_5	SO_3	Cl	K_2O	CaO	TiO_2	MnO	Fe_2O_3	ZnO	SrO	ZrO_2	WO_3	PbO
**Poplar bark**	0	0.16	0	0.03	0.15	0.09	0	0.3	2.57	0	0.03	0.04	0	0.02	0	0	0
**Straw**	0	0	0	0	0	0	0.38	1.94	0.93	0	0.01	0.07	0.01	0	0	0.11	0
**Cassava peel**	0	0.02	0.02	0	0	0.08	0.06	0.55	0.67	0.07	0.06	0.25	0	0	0.02	0.03	0
**Almond shell**	0	0.03	0	0.07	0.04	0.03	0.01	0.38	0.34	0	0.01	0.23	0	0	0	0	0
**Orange peel**	0	0.04	0	0.05	0.07	0.09	0.03	0.95	1.26	0	0.01	0.02	0	0.03	0	0	0
**Mixed wood chip**	0	0.04	0	0	0.16	0	0.03	0.28	3.4	0	0.19	0.21	0.04	0	0	0.22	0.02
**Hemp**	0	0	0	0	0	0.02	0.07	0.65	1.24	0	0.01	0.03	0	0	0	0.05	0
**Chestnut dust**	0	0.02	0.01	0.13	0.01	0.03	0	0.04	0.26	0.01	0.02	0.2	0	0	0	0	0
**Cobnut shell**	0	0.04	0	0.1	0.03	0.05	0.01	0.42	0.5	0	0.02	0.06	0	0	0	0	0
**Pomegranate shell**	NA	NA	NA	NA	NA	NA	NA	NA	NA	NA	NA	NA	NA	NA	NA	NA	NA

Table S2: Oxide composition of cement and biomass ashes (% w/w)

Type of biomass	Cement/Bio-mass Ash	K_2O	CaO	SO_3	MgO	SiO_2	P_2O_5	Al_2O_3	Na_2O	Fe_2O_3	Cl	SrO	MnO	TiO_2	ZnO
	Cement (CEM1)	0.86	55.22	4.28	0.66	12.7	0.13	2.91	0.26	2.74	0.074	0.09	0.05	0.20	0.04
**Wood**	Mixed wood chip	6.00	53.6	3.75	5.49	10.08	8.52	0.96	0.27	0.65	-	0.07	1.16	0.06	0.10
	Poplar shavings	4.14	75.02	1.04	2.88	1.07	0.93	0.14	0.36	0.19	0.09	0.09	0.04	-	0.32
	Chestnut saw dust	6.28	45.37	2.0	3.2	12.1	1.52	1.5	0.21	2.80	-	0.075	0.88	0.165	0.32
**Shell**	Hazelnut shell	21.16	18.14	1.1	3.63	8.81	2.77	1.07	0.40	1.51	0.12	0.045	0.44	0.114	0.027
	Almond shell	24.43	24.13	14.38	3.4	4.17	3.82	1.15	0.98	0.68	-	0.48	0.08	0.04	0.02
**Fibre**	Jute fibre	10.21	45.38	2.55	2.64	5.21	2.14	0.66	0.25	1.16	-	0.099	0.09	-	0.04
	Straw (wheat)	23.67	11.22	3.1	1.26	26.01	1.78	1.11	1.12	0.42	0.39	0.034	0.14	-	0.03
	Pomegranate	23.57	15.59	6.27	3.73	1.63	1.36	0.54	0.50	0.38	0.24	0.196	0.04	0.041	0.03
	Orange	21.78	38.83	3.92	3.7	3.15	4.35	0.81	1.14	0.29	-	0.22	0.05	-	0.032
	Cassava peel	6.37	37.77	3.35	16.35	4.42	2.69	0.28	0.78	0.48	-	0.20	0.53	-	0.12

Table S3: CO_2 uptake in biomass ashes after 4 carbonation cycles

Type	Biomass ash	CO_2 uptake (g/kg)				Total CO_2 uptake (% w/w)
		1 hr	1 hr	1 hr	24 hrs	
**Wood**	Mixed wood chip	26.2	248.7	260.8	262.7	26.3
	Poplar shavings	70.6	87.5	254.1	414.0	41.4
	Chestnut saw dust	92.1	94.9	109.9	186.3	18.6
**Shell**	Hazelnut shell	62.35	64.4	82.9	113.7	11.4
	Almond shell	69.7	82.22	84.11	155.6	15.6
**Fibre**	Jute fibre	188.0	216.6	218.5	294.5	29.4
	Straw (wheat)	17.0	36.3	37.0	48.6	4.9
**Fruit peel**	Cassava	86.7	99.9	103.1	127.2	12.7
	Pomegranate	87.4	87.4	101	135.8	13.6
	Orange	67.3	104.9	149.6	150.0	15.0

Fig. S1 (a)

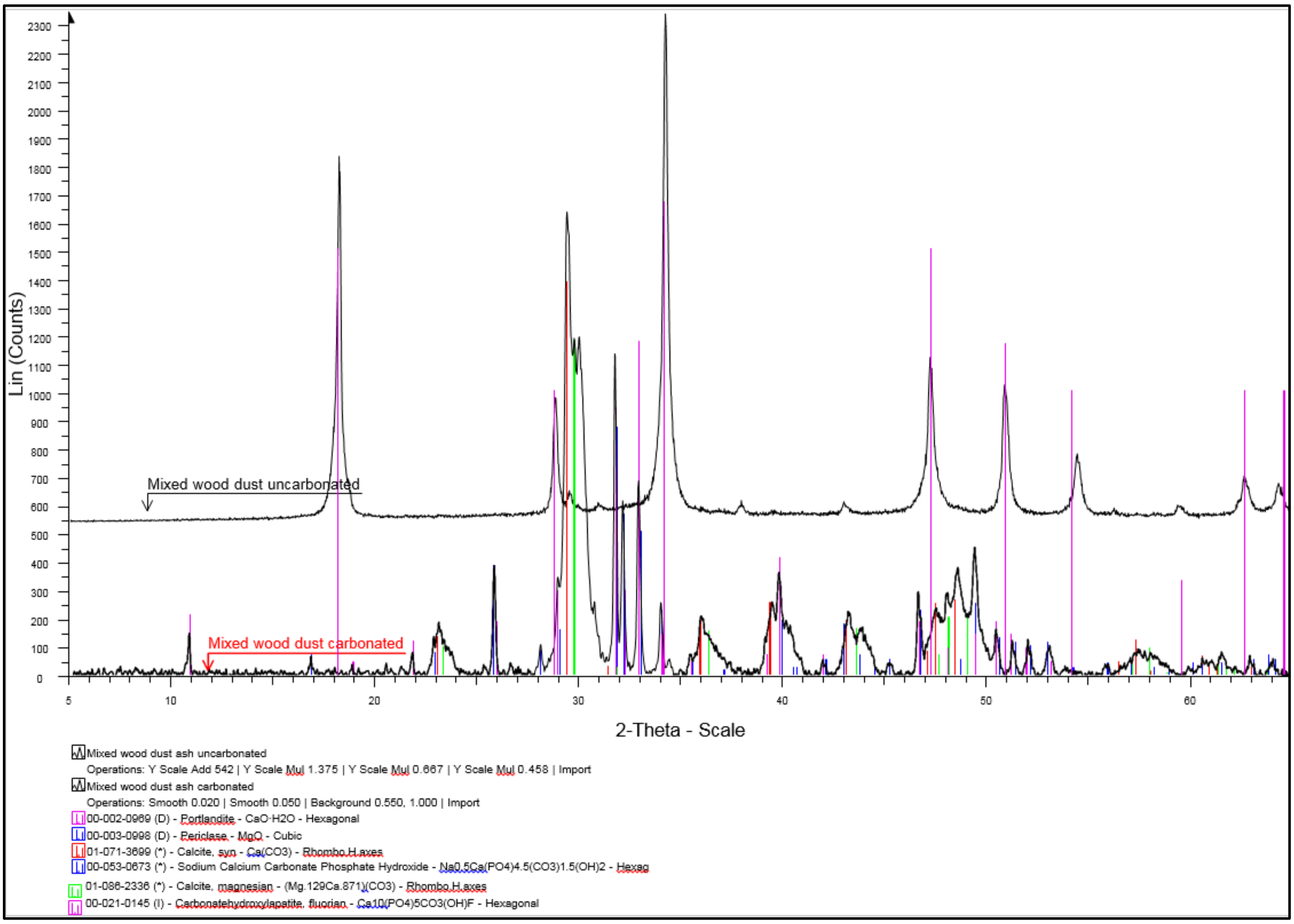
Lin (Counts)
2-Theta - Scale
Mixed wood dust uncarbonated
Mixed wood dust carbonated
Mixed wood dust ash uncarbonated
Operations: Y Scale Add 542 | Y Scale Mul 1.375 | Y Scale Mul 0.667 | Y Scale Mul 0.458 | Import
Mixed wood dust ash carbonated
Operations: Smooth 0.020 | Smooth 0.050 | Background 0.550, 1.000 | Import
00-002-0969 (D) - Portlandite - CaO·H2O - Hexagonal
00-003-0998 (D) - Periclase - MgO - Cubic
01-071-3699 (*) - Calcite, syn - Ca(CO3) - Rhombo.H.axes
00-053-0673 (*) - Sodium Calcium Carbonate Phosphate Hydroxide - Na0.5Ca(PO4)4.5(CO3)1.5(OH)2 - Hexag
01-086-2336 (*) - Calcite, magnesian - (Mg.129Ca.871)(CO3) - Rhombo.H.axes
00-021-0145 (I) - Carbonatehydroxylapatite, fluorian - Ca10(PO4)5CO3(OH)F - Hexagonal

Fig. S1(b)

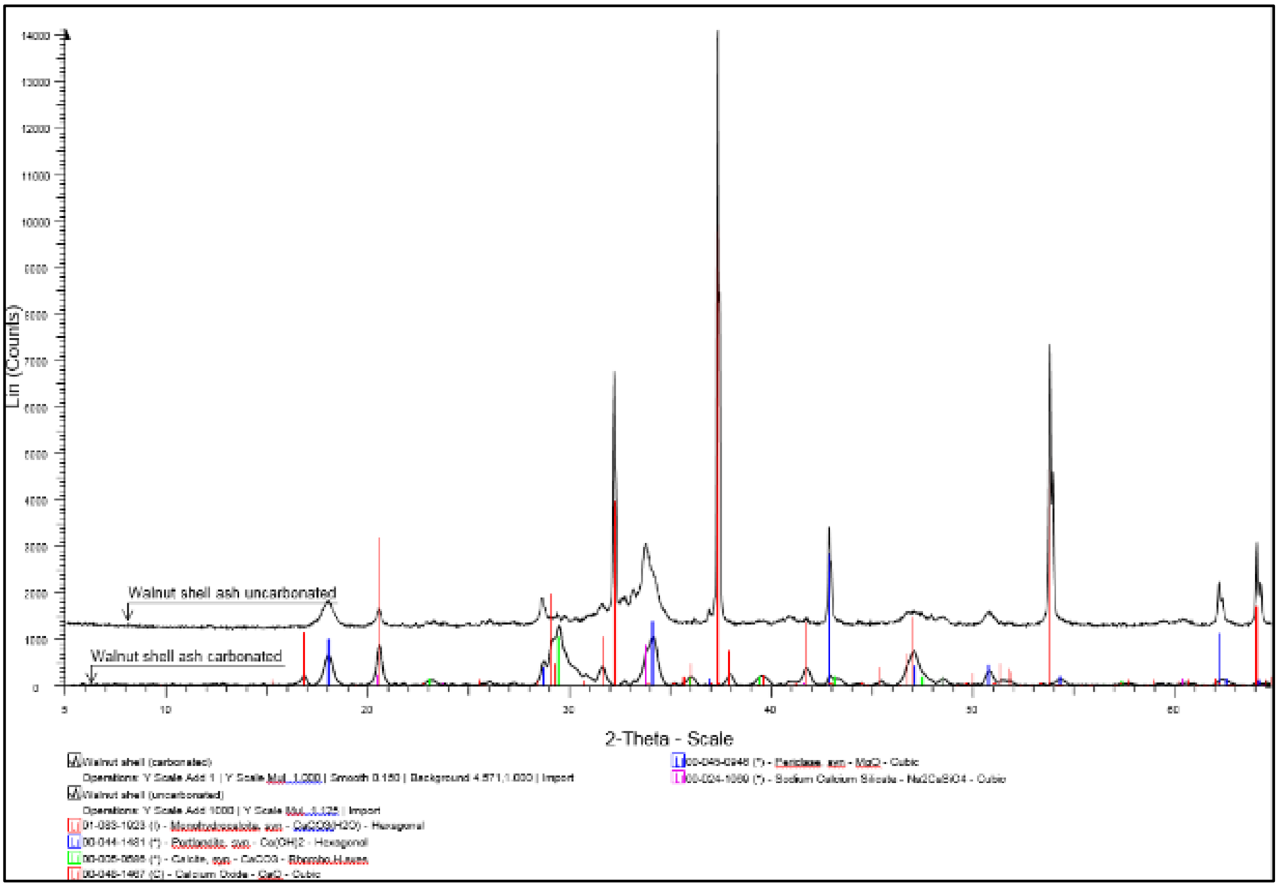
Lin (Counts)
2-Theta - Scale
Walnut shell ash uncarbonated
Walnut shell ash carbonated
Walnut shell (carbonated)
Walnut shell (uncarbonated)
Portlandite, syn - Ca(OH)2 - Hexagonal
Calcium Oxide - CaO - Cubic
Periclase, syn - MgO - Cubic
Sodium Calcium Silicate - Na2CaSiO4 - Cubic

Fig. S1(c)

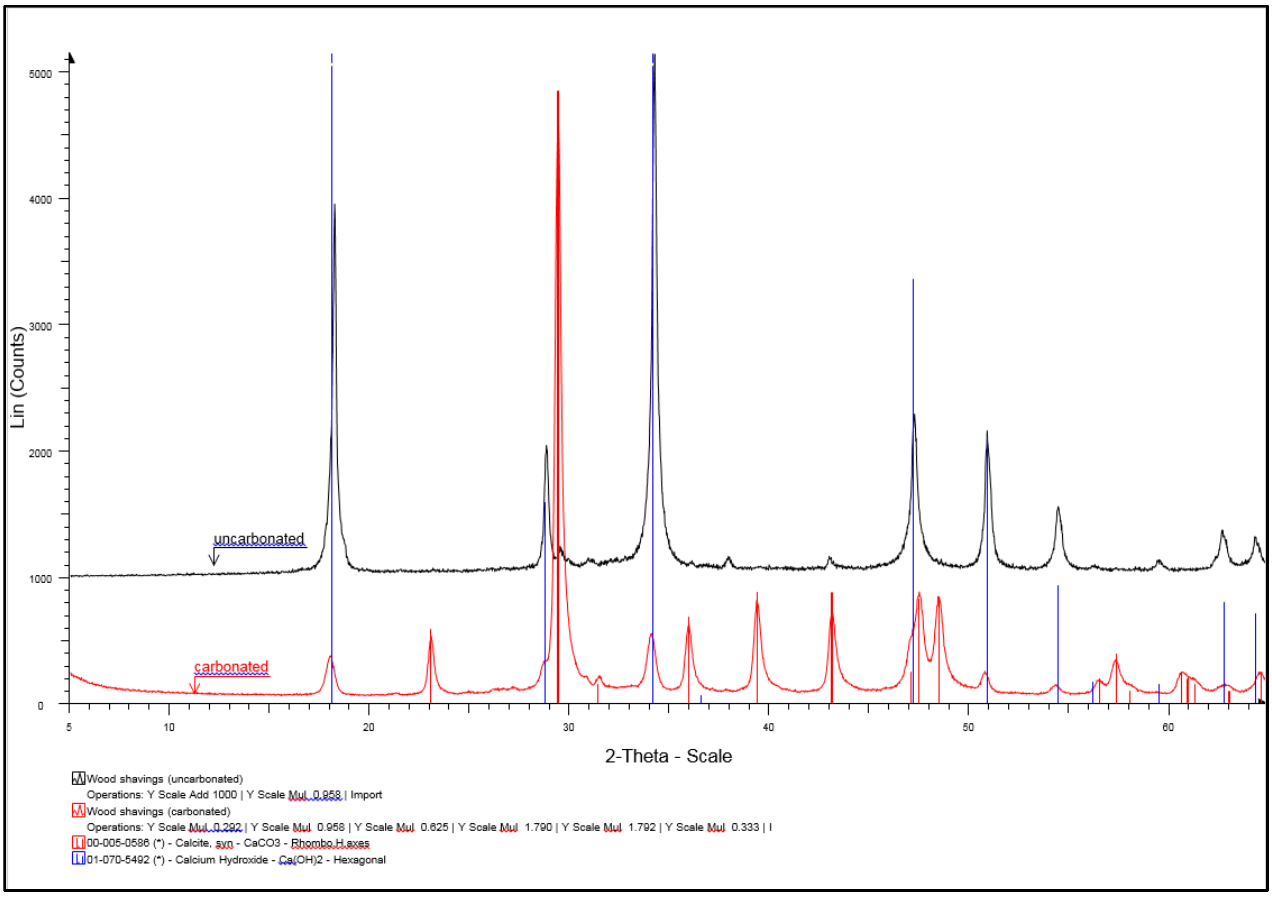

Fig. S1: X-ray diffractograms of biomass ash residues before and after carbonation: (a) mixed wood dust, (b) nut shell, (c) wood shavings. Calcium oxide, portlandite, and calcite are indicated.

Type (i)	Type (ii)	Type (iii)
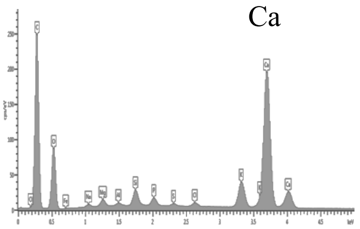	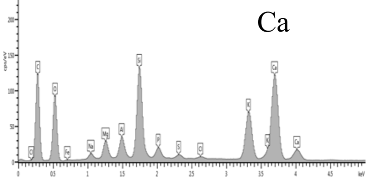	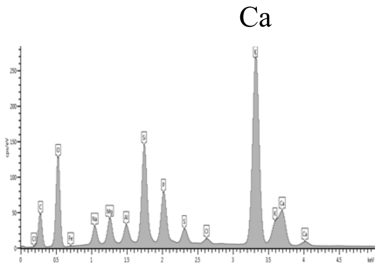

Fig. S2: EDS Spectra of carbonated biomass ash cylinders highlighting calcium (shown in Fig. 2 in MSS)

Fig. S3 (a)

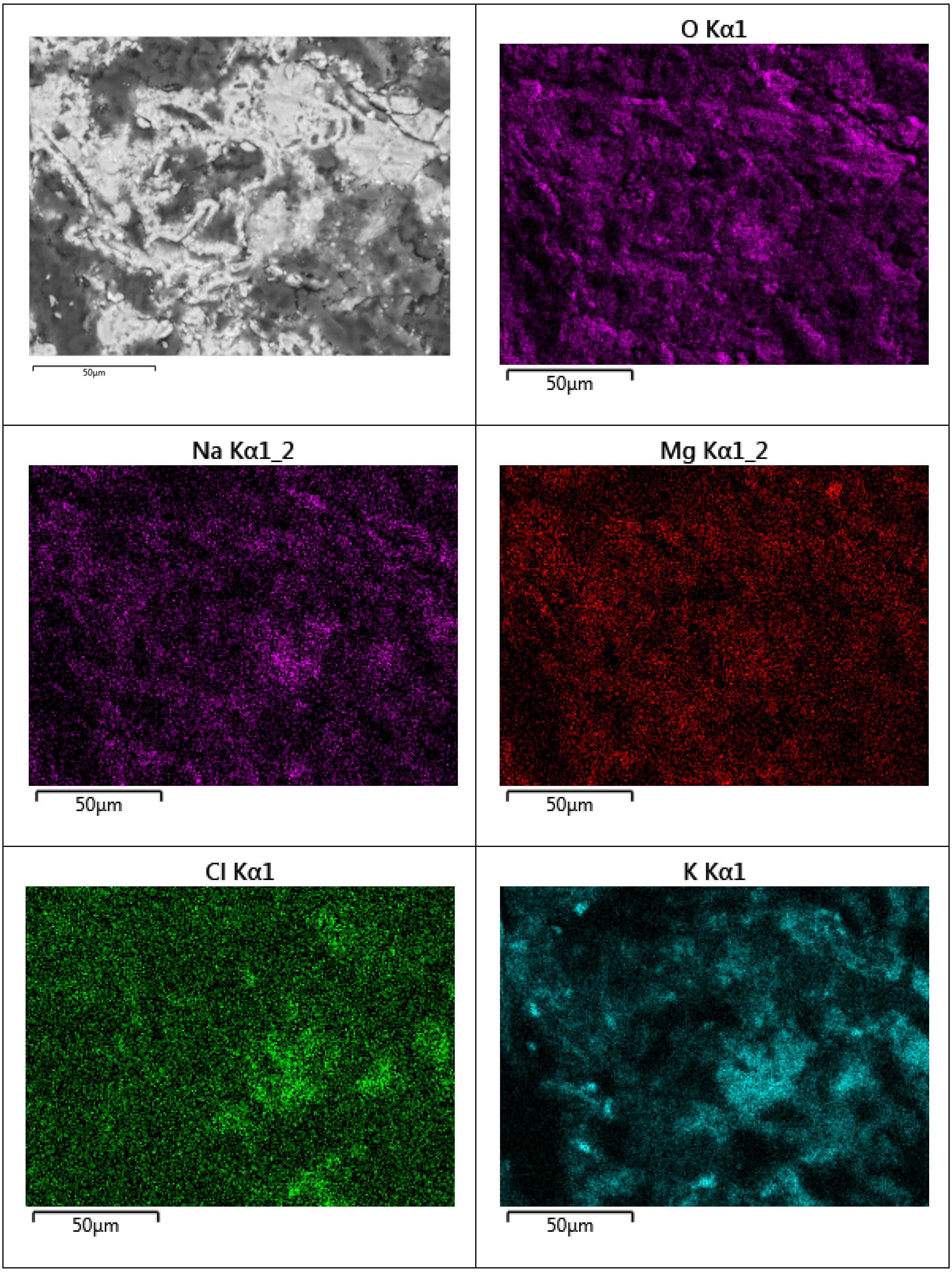

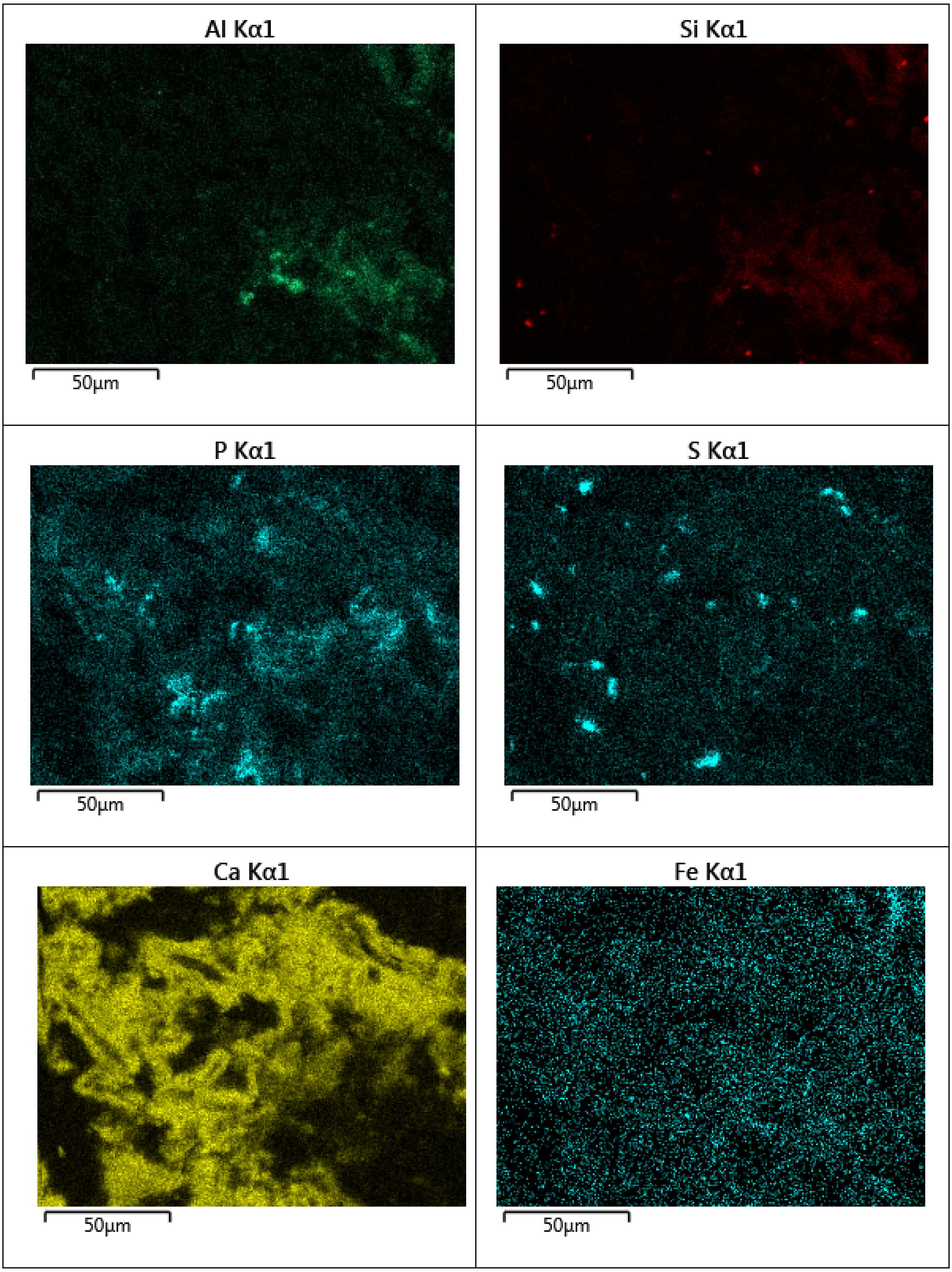
Al Kα1
50µm
Si Kα1
50µm
P Kα1
50µm
S Kα1
50µm
Ca Kα1
50µm
Fe Kα1
50µm

Fig. S3 (b)

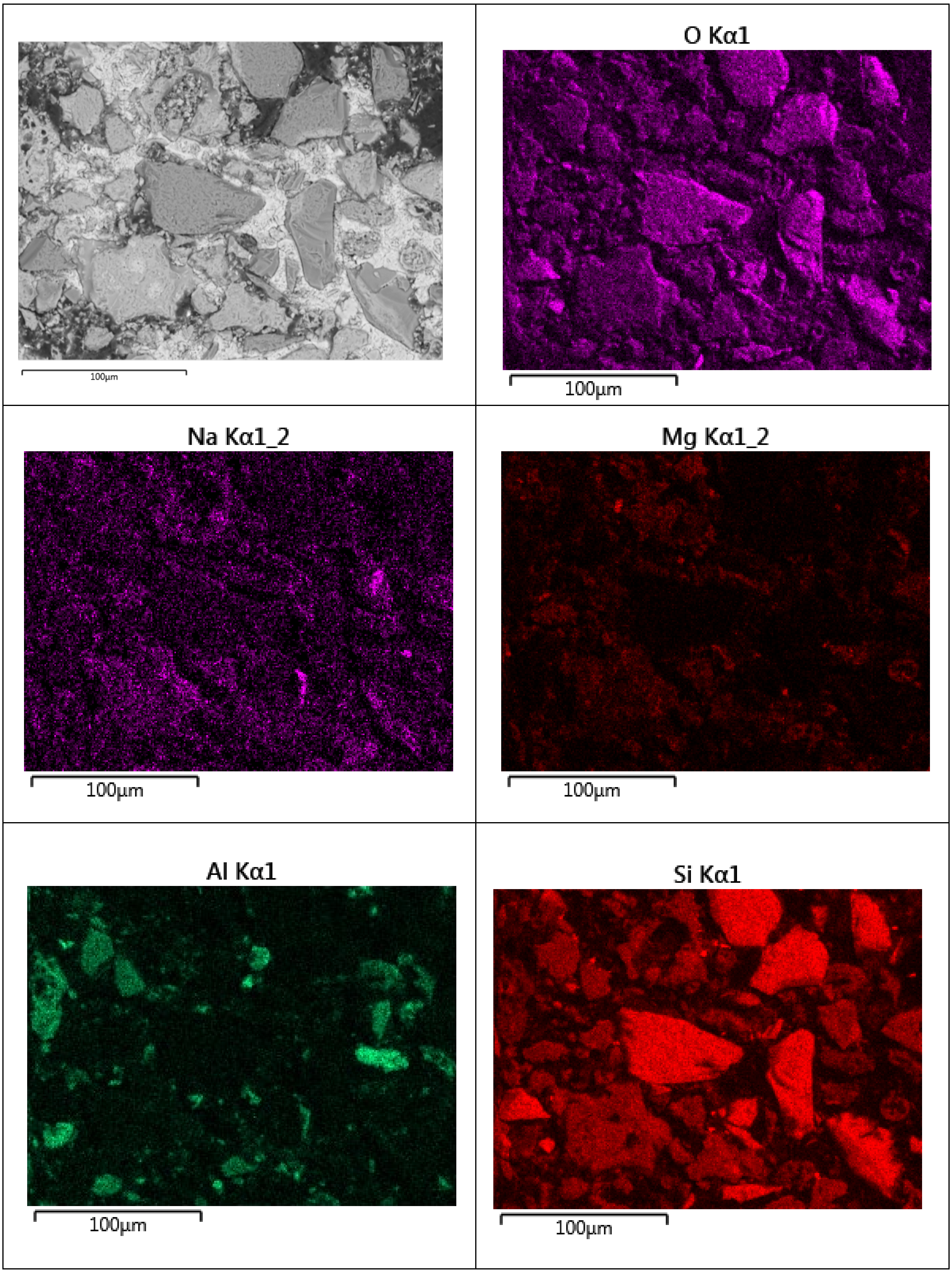
100µm
O Kα1
100µm
Na Kα1_2
100µm
Mg Kα1_2
100µm
Al Kα1
100µm
Si Kα1
100µm

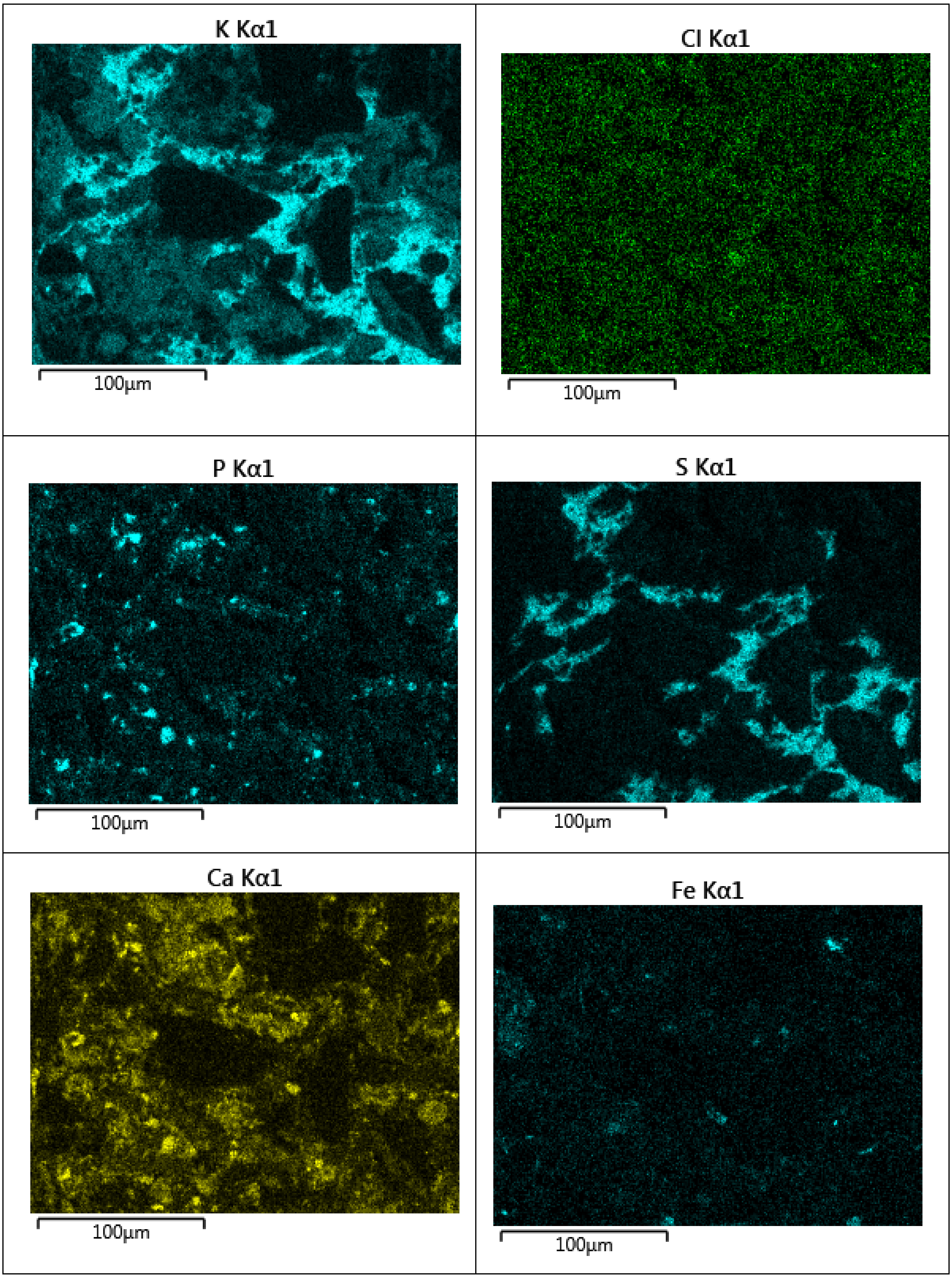
K Kα1
100µm
Cl Kα1
100µm
P Kα1
100µm
S Kα1
100µm
Ca Kα1
100µm
Fe Kα1
100µm

Fig. S3 (c)

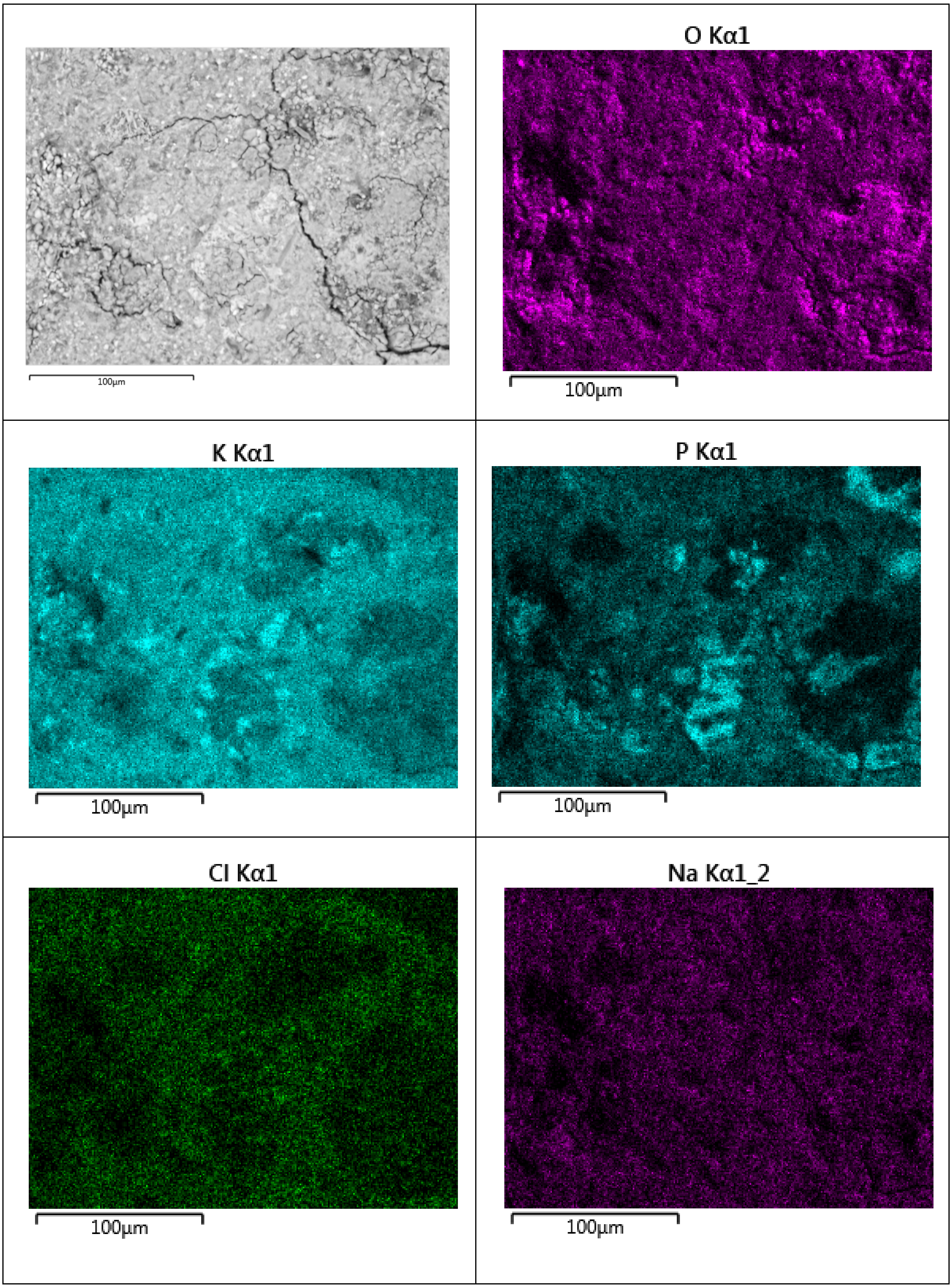
100µm
O Kα1
100µm
K Kα1
100µm
P Kα1
100µm
Cl Kα1
100µm
Na Kα1_2
100µm

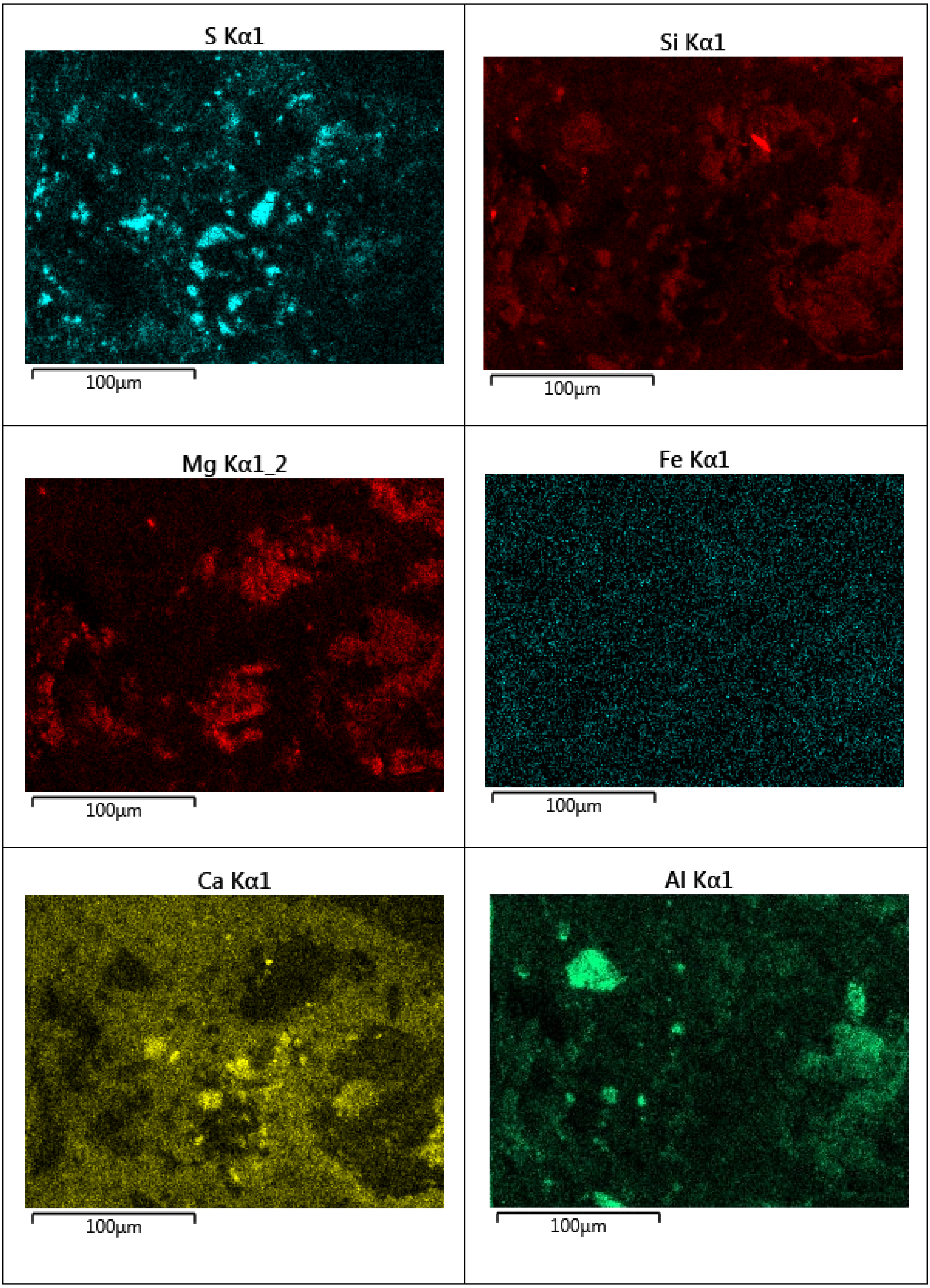

Fig. S3: Elemental analysis (EDS) of micrographs of carbonated biomass ash cylinders as shown in figure 2 (a) - Mixed wood ash, showing relic-planty structures; (b) - Nut shell-derived ash, rich in silica; (c) - Wood ash, with dispersed, discrete precipitates of carbonate
